# Supplementary material for: Liposomal Irinotecan for Treatment of Colorectal Cancer in a Preclinical Model
Source: Cancers (Basel). 2019 Feb 27;11(3):281. doi: 10.3390/cancers11030281 (PMC6468623; doi:10.3390/cancers11030281)
Supplement: Supplementary file 1 [file cancers-11-00281-s001.pdf]

Supplementary Materials: Liposomal Irinotecan for Treatment of Colorectal Cancer in a Preclinical Model

Jiao-Ren Huang, Mei-Hsien Lee, Wen-Shan Li and Han-Chun Wu

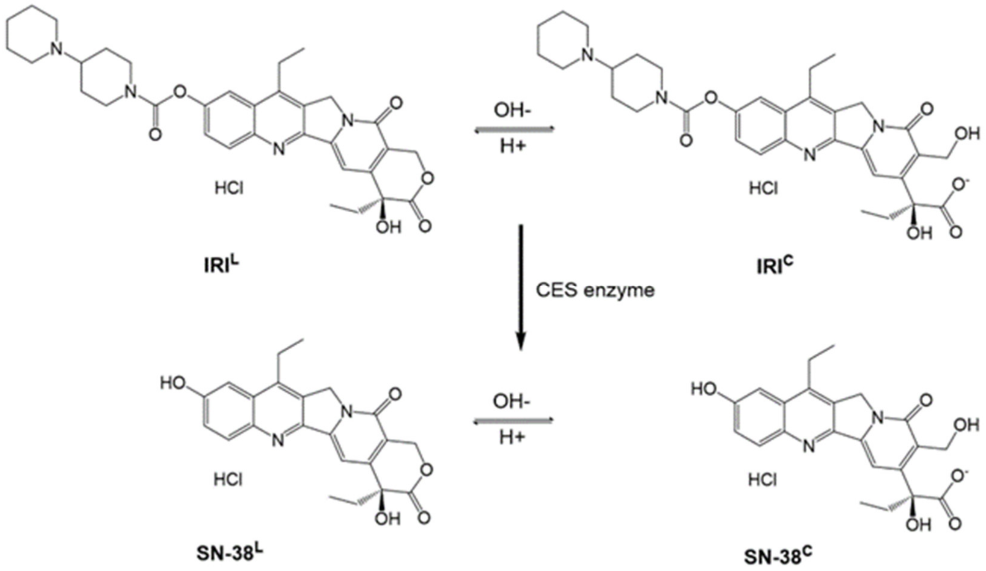

Figure S1. The structures of irinotecan and SN-38. Irinotecan (IRI) exists in a pH-dependent reversible equilibrium between closed active lactone and open inactive carboxylate conformations. The lactone form of IRI (IRI^L) is predominant in acidic conditions, whereas the carboxyl form of IRI (IRI^C) exists mainly in basic environments. SN-38 is a major metabolite of IRI, which is converted by carboxylesterase (CES) enzyme.

A

IC_{50} [μM]	IRI	SN-38
A549	36.39	1.475
CL1-5	47.01	0.196
SK-HEP-1	13.58	3.672
Ca9-22	43.17	0.110
HCT 116	25.37	0.175
PC-3	76.81	0.634
MCF-7	129.08	0.510
MIA PaCa - 2	58.28	0.444

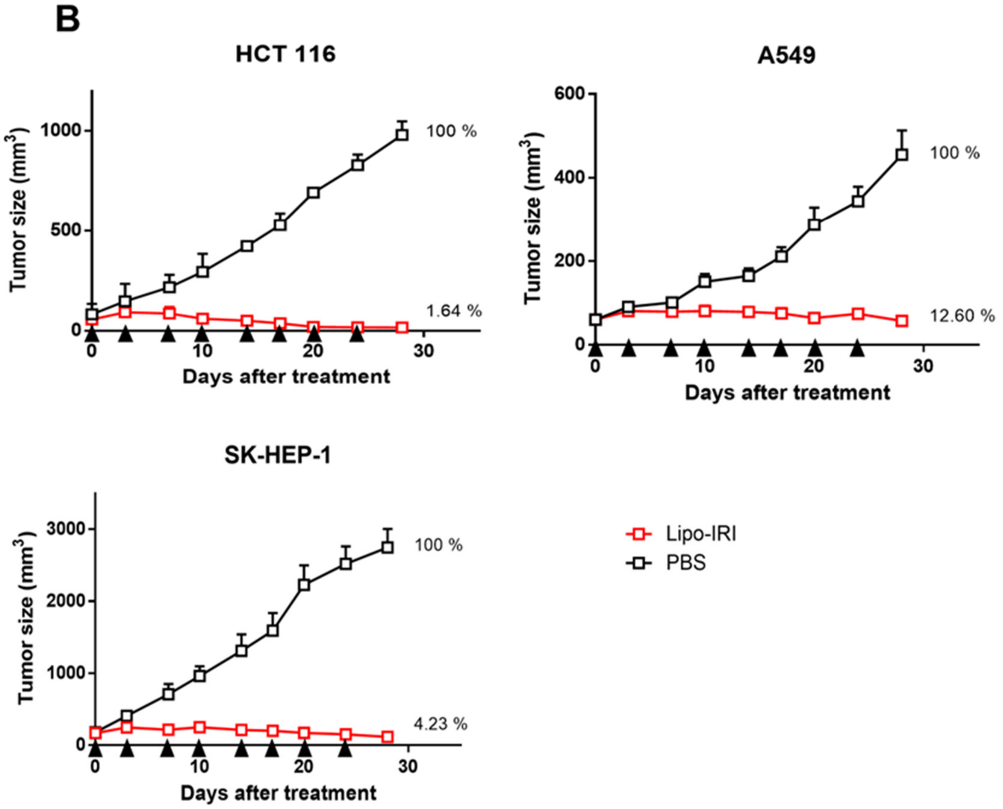

Figure S2. Cytotoxicity in vitro and tumor response in vivo. (**A**) Cell viability was evaluated by MTT assay. IC_{50} values were calculated with GraphPad Prism 6.0 software. (**B**) NOD/SCID mice were inoculated with HCT 116, A549 or SK-HEP-1cell lines. Tumor-bearing mice received 10 mg/kg Lipo-IRI i.v. through the tail vein twice per week.

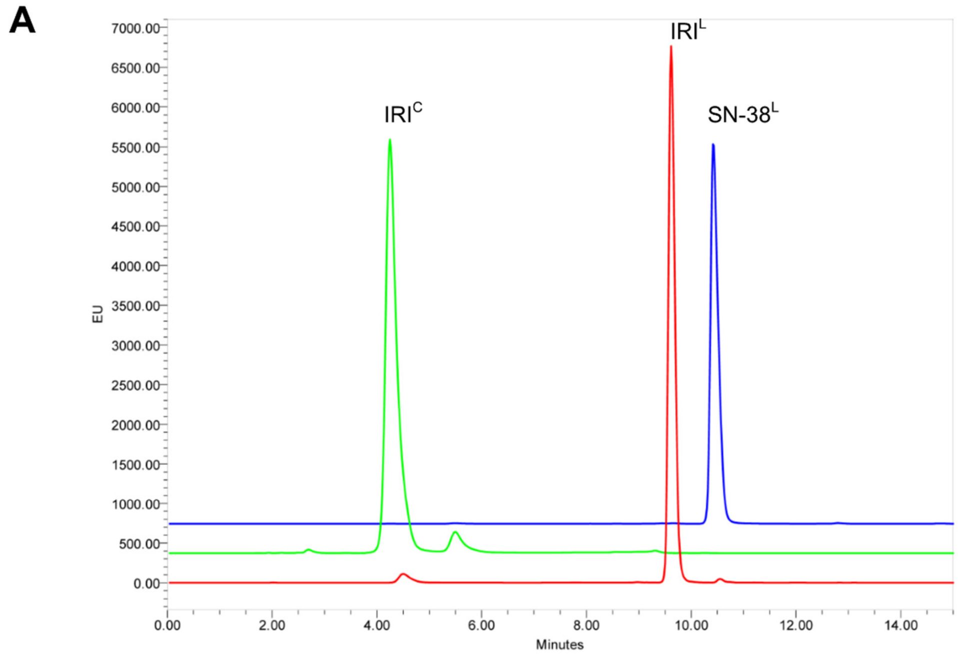

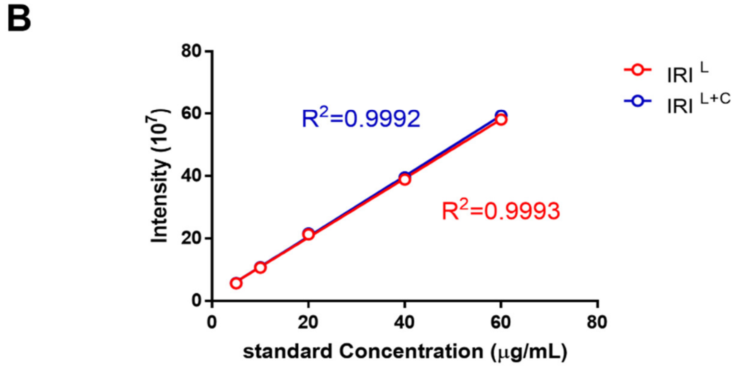

Figure S3. Chromatogram of IRI and SN-38. (**A**) HPLC separation of IRI lactone and carboxylate species. Chromatography conditions were as follows. Mobile phase: ACN-TEAA (3%, pH 5.5.) from 20:80 to 70:30 in 15 min. Flow rate: 0.9 mL/min. Fluorescence detector was operated at excitation 375 nm and emission 500 nm. IRI^L, IRI^C and SN-38^L are shown as red, green, and blue lines, respectively. (**B**) A calibration curve for the IRI standard was analyzed and plotted. R^2 = 0.993 for the lactone and 0.992 for total.

© 2019 by the authors. Licensee MDPI, Basel, Switzerland. This article is an open access article distributed under the terms and conditions of the Creative Commons Attribution (CC BY) license (http://creativecommons.org/licenses/by/4.0/).
